# Editorial: Sepsis: basic, clinical and therapeutic approaches, volume II

**DOI:** 10.3389/fphar.2023.1339116

**Published:** 2023-12-06

**Authors:** Hong Zhou, Yan Kang, Daolin Tang, Lefu Lan

**Affiliations:** ^1^ Key Laboratory of Basic Pharmacology of Ministry of Education and Joint International Research Laboratory of Ethnomedicine of Ministry of Education, Zunyi Medical University, Zunyi, Guizhou, China; ^2^ Department of Critical Care Medicine, Institute of Critical Care Medicine, West China Hospital, Sichuan University, Chengdu, Sichuan, China; ^3^ West China Tianfu Hospital, Sichuan University, Chengdu, Sichuan, China; ^4^ Department of Surgery, UT Southwestern Medical Center, Dallas, TX, United States; ^5^ School of Pharmaceutical Science and Technology, Hangzhou Institute for Advanced Study, University of Chinese Academy of Sciences, Hangzhou, China

**Keywords:** sepsis, immune disorder, multiple organ dysfunction, drugs, evaluation

Sepsis is a life-threatening organ dysfunction caused by the host’s uncontrolled response to infection, impacting millions of people around the world and killing between one in three and one in six of those it affects each year (Evans et al., 2021). The pathogenesis of sepsis is very complex, involving multiple systems and organs. The immune system plays a core role in sepsis, presenting as an early cytokine storm and late immunosuppression. Till now sepsis treatment adopts comprehensive treatment, and there is no effective specific drug to balance immune reaction (Evans et al., 2021). Here, we focus developing new drugs and drug targets to combat sepsis and provide a new direction for sepsis drugs investigation.

As well-known, the sepsis immune response is not a local and transient process but a complex and continuous process involving all major cell types of innate and adaptive immunity (van der Poll, et al., 2021). B cells are traditionally studied for their ability to produce antibodies in the context of mediating humoral immunity, but they are immune cells that have been neglected in sepsis studies. To one’s relief, over the past few years, B cells have been increasingly recognized as key modulators of adaptive and innate immunity during sepsis, and they can participate in immune responses by presenting antigens, producing cytokines, and modulating other immune cells. Recently, increasing evidence links B-cell dysfunction to mechanisms of immune derangement in sepsis, which has drawn attention to the powerful properties of this unique immune cell type in sepsis. Herein, Ma et al. reviewed the dynamic alterations of B cells and their novel roles in animal models and patients with sepsis, and provided new perspectives for therapeutic strategies targeting B cells in sepsis.

Sepsis is a multi-organ dysfunction syndrome caused by anomalous host response to infection, in which kidney injury is closely related to the progression and outcome of sepsis patients. There are many factors involved in the pathological process of acute kidney injury, such as reactive oxygen species (ROS), endoplasmic reticulum stress, and epithelial-mesenchymal transition.

In the past literature, melatonin (N-acetyl-5-methoxytryptamine; MLT) has been shown to have a renalprotective effect against kidney injury. Herein, Chen et al. investigated the mechanisms underlying the protective role of MLT in sepsis-induced renal injury. The results showed MLT alleviated renal dysfunction with the increase of blood urea nitrogen and serum creatinine and reduction of fibrosis in mice cecal ligation puncture (CLP) model. RNA-seq analysis showed that MLT repressed the oxidant stress in response to kidney injury. *In vitro* study showed that MLT suppresses lipopolysaccharide-induced accumulation of ROS production. Taken together, the results showed that MLT alleviated renal damage by regulating the production of ROS.

Preclinical experiments initially reveal the effectiveness of a drug, but whether it works in humans needs to be evaluated through standardized clinical trials. Sodium bicarbonate solution (SB) is commonly applied in fluid resuscitation and correcting the disorders of acid and base in the early stage after sepsis, and SB therapy was widely investigated for its relationship with mortality in severe septic patients (Yagi and Fujii, 2021). In the update of the Surviving Sepsis Campaign guidelines 2021 (Evans et al., 2021), it suggested against using SB treatment to make improvement in haemodynamics or to decrease the consumption of vasopressor for adults with septic shock and lactic academia induced by hypoperfusion. However, the effect of SB therapy on death rate when used in most septic patients with moderate lactic acidosis (MLA) is still unknown. Herein, Huang et al. retrospectively analyzed data from 512 septic patients with acute MLA identified from the large ICU database. The analysis results demonstrated that SB therapy could reduce ICU and hospital mortality of septic patients with acute MLA. Meanwhile, it could also improve hospital survival in the subgroup of septic shock patients with acute MLA. Of course, randomized controlled clinical trials are further required to validate the conclusions.

In preclinical experiments, granisetron, the 5-HT_3_ receptor antagonist, was demonstrated to reduce inflammation and improved survival of septic mice. Herein, Guan et al. reported that a randomized controlled trial was designed to assess the efficacy and safety of granisetron in patients with sepsis. The modified intention-to-treat analysis included 150 septic patients. The 28-day all-cause mortalities in the granisetron and placebo groups were 34.7% and 35.6%, respectively. No differences were observed in secondary outcomes. In the subgroup analysis of patients without abdominal or digestive tract infections, the 28-day mortality in the granisetron group was 10.9% lower than mortality in the placebo group. Adverse events were not statistically different between the groups. The results demonstrated granisetron did not improve 28-day mortality in patients with sepsis, but a further clinical trial targeting septic patients without abdominal/digestive tract infections perhaps is worthy of consideration.

Tuberculosis caused by *mycobacterium tuberculosis* is a serious threat to human health. New antimicrobial strategy plays a vital role in the treatment of tuberculosis caused by drug-resistant tuberculosis. The treatment of drug-resistant tuberculosis usually requires a combination of new anti-tuberculosis drugs (WHO, 2022). Now, a new, effective anti-tuberculosis regimen containing bedaquiline (BDQ) and pyrifazimine (TBI-166) has been recommended for a phase IIb clinical trial. Preclinical drug-drug interaction studies of the combination of BDQ and TBI-166 have been designed to support future clinical trials. Herein, Ding et al. investigated whether a drug-drug interaction between BDQ and TBI-166 affects the pharmacokinetics of BDQ. The results showed that the combination of BDQ and TBI-166 significantly reduced exposure to the toxic metabolite M2 by inhibiting the activity of the CYP3A4 pathway. The potential safety and efficacy benefits demonstrated by the TB treatment highly suggest that coadministration of BDQ and TBI-166 should be investigated further.

In summary, the Research Topic of aforementioned articles in this Research Topic provides either overview of novel targets in immunotherapy, or new fundamental findings and summaries related to sepsis. However, it is of great significance to discover new drug targets based on the pathophysiological mechanism of sepsis and evaluate the data from existing drug and new combination of drugs in clinical trials.
